# Differential association between the *GLP1R* gene variants and brain functional connectivity according to the severity of alcohol use

**DOI:** 10.1038/s41598-022-17190-3

**Published:** 2022-07-29

**Authors:** Mehdi Farokhnia, Samantha J. Fede, Erica N. Grodin, Brittney D. Browning, Madeline E. Crozier, Melanie L. Schwandt, Colin A. Hodgkinson, Reza Momenan, Lorenzo Leggio

**Affiliations:** 1 Clinical Psychoneuroendocrinology and Neuropsychopharmacology Section, Translational Addiction Medicine Branch, National Institute on Drug Abuse Intramural Research Program and National Institute On Alcohol Abuse and Alcoholism Division of Intramural Clinical and Biological Research, National Institutes of Health, Baltimore, MD USA; 2 Center on Compulsive Behaviors, National Institutes of Health, Bethesda, MD USA; 3 Department of Mental Health, Johns Hopkins Bloomberg School of Public Health, Johns Hopkins University, Baltimore, MD USA; 4 Clinical NeuroImaging Research Core, National Institute on Alcohol Abuse and Alcoholism Division of Intramural Clinical and Biological Research, National Institutes of Health, Bethesda, MD USA; 5 Department of Psychology, University of California, Los Angeles, Los Angeles, CA USA; 6 Office of the Clinical Director, National Institute on Alcohol Abuse and Alcoholism Division of Intramural Clinical and Biological Research, National Institutes of Health, Bethesda, MD USA; 7 Laboratory of Neurogenetics, National Institute on Alcohol Abuse and Alcoholism Division of Intramural Clinical and Biological Research, National Institutes of Health, Rockville, MD USA; 8 Medication Development Program, National Institute on Drug Abuse Intramural Research Program, National Institutes of Health, Baltimore, MD USA; 9 Center for Alcohol and Addiction Studies, Department of Behavioral and Social Sciences, School of Public Health, Brown University, Providence, RI USA; 10 Division of Addiction Medicine, Department of Medicine, School of Medicine, Johns Hopkins University, Baltimore, MD USA; 11 Department of Neuroscience, Georgetown University Medical Center, Washington, DC USA

**Keywords:** Addiction, Risk factors

## Abstract

Growing evidence suggests that the glucagon-like peptide-1 (GLP-1) system is involved in mechanisms underlying alcohol seeking and consumption. Accordingly, the GLP-1 receptor (GLP-1R) has begun to be studied as a potential pharmacotherapeutic target for alcohol use disorder (AUD). The aim of this study was to investigate the association between genetic variation at the GLP-1R and brain functional connectivity, according to the severity of alcohol use. Participants were 181 individuals categorized as high-risk (*n* = 96) and low-risk (*n* = 85) alcohol use, according to their AUD identification test (AUDIT) score. Two uncommon single nucleotide polymorphisms (SNPs), rs6923761 and rs1042044, were selected a priori for this study because they encode amino-acid substitutions with putative functional consequences on GLP-1R activity. Genotype groups were based on the presence of the variant allele for each of the two GLP-1R SNPs of interest [rs6923761: AA + AG (*n* = 65), GG (*n* = 116); rs1042044: AA + AC (*n* = 114), CC (*n* = 67)]. Resting-state functional MRI data were acquired for 10 min and independent component (IC) analysis was conducted. Multivariate analyses of covariance (MANCOVA) examined the interaction between GLP-1R genotype group and AUDIT group on within- and between-network connectivity. For rs6923761, three ICs showed significant genotype × AUDIT interaction effects on within-network connectivity: two were mapped onto the anterior salience network and one was mapped onto the visuospatial network. For rs1042044, four ICs showed significant interaction effects on within-network connectivity: three were mapped onto the dorsal default mode network and one was mapped onto the basal ganglia network. For both SNPs, *post-hoc* analyses showed that in the group carrying the variant allele, high versus low AUDIT was associated with stronger within-network connectivity. No significant effects on between-network connectivity were found. In conclusion, genetic variation at the GLP-1R was differentially associated with brain functional connectivity in individuals with low versus high severity of alcohol use. Significant findings in the salience and default mode networks are particularly relevant, given their role in the neurobiology of AUD and addictive behaviors.

## Introduction

Alcohol use disorder (AUD) is a chronic relapsing brain disease and a major public health problem with considerable medical, psychosocial, and economic burden^1,2^. However, treatment options for AUD, including pharmacotherapies, are limited. A better understanding of the neurobiological processes involved in alcohol use is critical for developing additional effective treatments^3^. While most of the research to date has been geared toward central mechanisms of addictive behaviors and alcohol use, there is a growing interest in the role of peripheral pathways (e.g., endocrine systems, immune factors) and their communications with the brain. The biobehavioral overlap between alcohol use and feeding/metabolic pathways is particularly relevant, because alcohol is not only a drug with pharmacological actions in the periphery and in the central nervous system, but also is a source of calories, impacts metabolism, and is consumed as a palatable drink^4,5^. One hormone that links the gastrointestinal and central nervous systems, is involved in feeding and metabolism, and has begun to be investigated in relation to addictive behaviors including AUD, is glucagon-like peptide-1 (GLP-1)^6^.

GLP-1 is a 30-amino acid peptide hormone primarily produced by endocrine cells of the intestinal mucosa. The main function of GLP-1 includes regulation of glucose homeostasis and food intake via interaction with both peripheral (e.g., pancreas) and central (e.g., hypothalamus) receptors. In addition, GLP-1 acts as a neuropeptide and is synthesized by preproglucagon (PPG) neurons of the nucleus tractus solitarius (NTS). These neurons project to several brain regions involved in reward processing, such as the ventral tegmental area (VTA) and nucleus accumbens (NAc), suggesting a role in motivational behaviors^7^. The GLP-1 receptor (GLP-1R) is a G-protein coupled receptor (GPCR) expressed in various peripheral tissues, such as pancreatic β-cells, vagus nerve, and hepatic portal system, as well as brain regions, like hypothalamus, brainstem, globus pallidus, VTA, and NAc^8–12^. The GLP-1 system has been shown to modulate rewarding properties of food, alcohol, and other addictive drugs^13–16^. GLP-1 is also involved in stress regulation, mainly through interactions with the hypothalamic–pituitary–adrenal (HPA) axis^17–19^. Growing evidence from rodent experiments indicates that stimulation of GLP-1Rs attenuates alcohol-induced accumbal dopamine release, conditioned place preference for alcohol, alcohol drinking, and operant self-administration of alcohol^20–30^, providing evidence that the GLP-1 system plays a role in alcohol reward, seeking, and consumption, and should be further examined as a potential pharmacotherapeutic target. Nonetheless, human evidence in this regard is scarce^31^.

Neuroimaging studies in non-AUD healthy individuals demonstrate that endogenous GLP-1 levels, as well as exogenous GLP-1 administration, are associated with changes in brain activity in multiple regions, including NAc, amygdala, caudate, putamen, insula, anterior cingulate cortex (ACC), orbitofrontal cortex (OFC), prefrontal cortex (PFC), and hypothalamus^32–35^, most of which are implicated in AUD. Previous studies in humans have also examined genetic variation at the GLP-1R in relation to various metabolic and endocrine outcomes. Two missense single nucleotide polymorphisms (SNPs), namely rs6923761 and rs1042044, that result in amino-acid substitutions (rs6923761: glycine to serine at position 168, rs1042044: phenylalanine to leucine at position 260) and putative changes in the GLP-1R, have been found to be associated with outcomes such as anthropometric parameters, glucose tolerance, lipid profile, insulin levels, and cortisol levels^36–43^. Our group previously reported that the rs6923761 risk allele was associated with higher risk of AUD, greater alcohol administration and breath alcohol measures in an intravenous alcohol self-administration experiment, and higher functional magnetic resonance imaging (fMRI) blood oxygen level dependent (BOLD) response in globus pallidus during a monetary incentive delay (MID) task^30^.

The aim of the present study was to investigate the association between genetic variation at the GLP-1R and resting-state brain functional connectivity, according to the severity of alcohol use. Of note, a recent study found that among various neuroimaging modalities including structural MRI, resting-state fMRI, task-based fMRI, and combined MRI features, resting-state connectivity best predicts alcohol use severity ^44^, hence our focus on resting-state connectivity in this study. We hypothesized that genetic variation at the GLP-1R, specifically the two SNPs mentioned above (rs6923761 and rs1042044) that affect the protein structure/function and were selected a priori for this analysis, differentially correlate with brain functional connectivity in individuals with low versus high severity of alcohol use, and that these differences may contribute to the risk/severity of AUD.

## Methods

### Participants

Individuals were screened under National Institute on Alcohol Abuse and Alcoholism (NIAAA) screening protocols (98-AA-0009 and 14-AA-0181), which serve as an entry for all NIAAA clinical studies. Participants who qualified for and completed a neuroimaging protocol (14-AA-0080), and had complete behavioral, genetics, and neuroimaging data, were included in the present study (*N* = 181). All protocols were conducted at the National Institutes of Health (NIH) Clinical Center (Bethesda, MD, USA), approved by the appropriate NIH Institutional Review Board (IRB), and performed in accordance with relevant guidelines/regulations and the Declaration of Helsinki. Participants provided written consents before enrollment and were compensated for their time and participation. To be included in the neuroimaging protocol (14-AA-0080), participants had to be: (A) 18 years of age or older, and (B) enrolled in the NIAAA screening protocol or determined eligible for another NIAAA study. Exclusion criteria were as follows: presence of ferromagnetic objects in the body that are contraindicated for brain MRI, fear of closed spaces, inability to lie comfortably flat on back for up to 2 h in the MRI scanner, pregnancy, left handedness, and/or symptoms of alcohol withdrawal, as indicated by a Clinical Institute Withdrawal Assessment for Alcohol, revised (CIWA-Ar) score of ≥ 8. All participants had to have a breath alcohol concentration (BrAC) of 0 to be enrolled and scanned.

### Behavioral and genetics data

As part of the screening, a comprehensive medical and psychiatric evaluation was performed, and blood samples were collected for DNA extraction and genetic analysis. To assess the severity of alcohol use, participants completed the Alcohol Use Disorders Identification Test (AUDIT), a validated and widely used 10-item self-reported measure that asks questions about alcohol consumption, drinking behaviors, and alcohol-related problems^45,46^. Items are scored on a scale from 0 (least severe) to 4 (most severe) and a total AUDIT score is calculated. Consistent with Conigrave and colleagues ^47^, and in order to examine a 2 × 2 interaction effect (see below), participants were categorized as high-risk/hazardous alcohol use (high-AUDIT, defined as a total score ≥ 8, *n* = 96, 53%) and low-risk/non-hazardous alcohol use (low-AUDIT, defined as total score < 8, *n* = 85, 47%).

Genotyping was performed at the NIAAA Laboratory of Neurogenetics. Genomic DNA was extracted from whole blood using standard protocols and genotyped using the genome-wide Illumina OmniExpress BeadChip array (Illumina, San Diego, CA, USA). Ancestry informative markers (AIMs) were also extracted from the Illumina array and ancestral proportions were calculated for all participants ^48^. Following a dominant model, participants were categorized into two genotype groups according to the presence of the mutant/risk allele for each of the two GLP-1R SNPs of interest. For rs6923761, the two groups were A-allele carriers (AA + AG, *n* = 65) and non-A-allele carriers (GG, *n* = 116). For rs1042044, the two groups were A-allele carriers (AA + AC, *n* = 114) and non-A-allele carriers (CC, *n* = 67).

### Neuroimaging data acquisition, preprocessing, and analysis

All brain images were acquired by a Siemens 3 T Skyra MRI machine (Siemens Medical Solutions USA, Inc., Malvern, PA). The resting-state functional MRI (rs-fMRI) was performed as part of a larger neuroimaging study that included structural, diffusion tensor, and task-based fMRI. The rs-fMRI scan lasted 10 min during which participants were instructed to stay awake while lying on their back in the dark with their eyes open and no additional stimuli. The structural scan was acquired using a T1-MPRAGE sequence (TR: 1900 ms, TE: 3.09 ms, flip angle: 10°, FOV:24 × 24 cm, 1 mm slice thickness, 144 slices, multi-slice mode: single shot). The rs-fMRI scan was acquired using an echoplanar-imaging pulse sequence (TR: 2000 ms, TE: 30 ms, flip angle: 90°, FOV: 24 × 24 cm, 38 × 38 × 38 mm^3^, 36 slices, multi-slice mode: interleaved).

The rs-fMRI data were preprocessed using Analyses of Functional Neuroimages (AFNI) v16.2.16 ^49^. Preprocessing was done on a single subject basis. For each time course, the first three TRs were removed and 3dDespike was applied to smooth spikes in signal. The time courses were then shifted for each voxel to be aligned to the same temporal origin by detrending, then interpolating the time series. Next, volumes spanning the time series were aligned to the base volume and the skull stripped anatomy of the participant, and then warped to standard Talairach space using the non-linear warping procedure 3dNwarpApply. Volumes were blurred with a 4-mm- full-width at half maximum Gaussian smoothing kernel. Individual data with an average motion derivative value of 0.3 mm/TR or higher, with > 3% of TRs being identified as above that cut-off, or with > 50 TRs having motion were removed prior to group level analysis. As part of the independent component analysis (ICA), which is detailed below, additional control for nuisance variables occurred through identification and extraction of signal due to motion or physiologic noise. Visual inspection of individual masks and registrations across modality were also performed, and if not acceptable, subjects were removed to ensure quality.

Following preprocessing, ICA was employed to analyze the rs-fMRI data in the Group ICA of fMRI Toolbox (GIFT) v3.0b in SPM 12. This is a data-driven signal processing approach that decomposes the rs-fMRI multivariate signal into subcomponents of networks with related fluctuating activity. We followed the steps outlined by Allen and colleagues^50^ for examining resting-state data which optimizes sensitivity, while reducing unnecessary testing. In this hierarchical approach, multivariate models are used to identify significant covariates and to facilitate testing predictors on the response matrices as a whole. Group ICA extracted 75 component time series, using a separation algorithm based on principal component analysis (PCA) at a single-subject level. Next, a group PCA was run to reduce the data to a single set of components for the group. Single-subject time series for each group component were back-reconstructed using the GICA3 algorithm. The resulting components were reviewed for stability using Icasso^51^, a software that compares component estimates iterated across multiple ICA runs. We also reviewed components manually to identify noise components. Of the 75 initial components, 24 were removed, and the remaining 51 were included in the statistical analysis, as described below. These 51 independent components (ICs) were labeled based on correlation with Resting State Network masks, using the Component Labeling toolbox within GIFT, and are listed in Table S1. Between component functional network connectivity (FNC) was also examined by correlating the time courses of each component with the rest of components, which resulted in a matrix of between-component connectivity for each subject.

### Statistical analysis

Demographic characteristics of the sample were summarized with descriptive statistics (mean and standard error for continuous variables, number and percent for categorical variables) and were compared between the two GLP-1R genotype groups for each SNP (independent samples t-test for continuous variables, chi-squared test for categorical variables). A similar comparison was performed between the two AUDIT groups. Our primary outcome of interest was the interaction between GLP-1R genotype and alcohol use severity on resting-state brain functional connectivity. To do so, multivariate analyses of covariance (MANCOVA) were run for each of the two GLP-1R SNPs, using the MANCOVAN toolbox within GIFT. MANCOVA models included GLP-1R genotype group (A-allele carrier versus non-A-allele carrier), AUDIT group (low-AUDIT versus high-AUDIT), and their interaction (GLP-1R genotype group × AUDIT group) as independent variables, age, sex (male or female), years of education, body mass index (BMI), smoking status (smoker or non-smoker), and AIMs scores (Europe and Africa) as covariates, and within- and between-network connectivity (spatial map intensity and FNC, respectively) as dependent variables^50^. For components with significant interactions in MANCOVA, connectivity estimates were extracted and graphed for visualization purposes. Significance level was set at *p* < 0.05 (2-tailed) for all analyses.

## Results

### Study sample

Table 1 presents demographic characteristics in the full sample and a comparison across the two GLP-1R genotype groups for each SNP. A similar comparison between the two AUDIT groups is presented in Table S2.

**Table 1:** Demographic characteristics of the full sample and stratified by genotype group.

	Full sample (*n* = 181)	rs6923761			rs1042044		
		GG (*n* = 116)	AA + AG (*n* = 65)	Statistics	CC (*n* = 67)	AA + AC (*n* = 114)	Statistics
Age, years, Mean (SEM)	40.57 (0.90)	39.97 (1.09)	42.15 (1.57)	*p* = 0.24	42.13 (1.58)	39.94 (1.08)	*p* = 0.24
**Years of education, Mean (SEM)**	14.95 (0.23)	14.72 (0.28)	15.35 (0.43)	*p* = 0.20	15.04 (0.37)	14.89 (0.30)	*p* = 0.76
**BMI, Kg/m**^**2**^**, Mean (SEM)**	26.72 (0.36)	26.64 (0.45)	26.85 (0.60)	*p* = 0.78	26.41 (0.54)	26.89 (0.48)	*p* = 0.52
**Sex, *n* (%)**							
Male	104 (57)	67 (58)	37 (57)	*p* = 0.91	39 (58)	65 (57)	*p* = 0.87
Female	77 (43)	49 (42)	28 (43)		28 (42)	49 (43)	
**Smoking status, *n* (%)**							
Smoker	47 (26)	28 (24)	19 (29)	*p* = 0.45	17 (25)	30 (26)	*p* = 0.88
Non-smoker	134 (74)	88 (76)	46 (71)		50 (75)	84 (74)	
**AIMs score, Mean (SEM)**							
Europe	0.50 (0.02)	0.37 (0.03)	0.73 (0.03)	*p* < 0.001	0.57 (0.04)	0.46 (0.03)	*p* = 0.07
Africa	0.37 (0.02)	0.47 (0.03)	0.18 (0.03)	*p* < 0.001	031 (0.04)	0.40 (0.03)	*p* = 0.15
**Race, *n* (%)**							
Black	76 (42)	63 (54)	13 (20)	*p* < 0.001	24 (35)	52 (46)	*p* = 0.18
White	77 (42)	31 (27)	46 (71)		36 (54)	41 (36)	
Asian	10 (6)	9 (8)	1 (1)		2 (3)	8 (7)	
Multiple races	9 (5)	6 (5)	3 (5)		2 (3)	7 (6)	
Unknown	9 (5)	7 (6)	2 (3)		3 (5)	6 (5)	
**90-day TLFB, Mean (SEM)**							
Average drinks per day	7.91 (0.67)	8.20 (0.88)	7.42 (1.00)	*p* = 0.57	8.17 (1.13)	7.76 (0.83)	*p* = 0.77
Heavy drinking days	30.56 (2.68)	31.29 (3.32)	29.26 (4.57)	*p* = 0.71	31.09 (4.46)	30.25 (3.37)	*p* = 0.88
**AUDIT group, *n* (%)**							
Low-AUDIT	85 (47)	54 (47)	31 (48)	*p* = 0.88	34 (51)	51 (45)	*p* = 0.43
High-AUDIT	96 (53)	62 (53)	34 (52)		33 (49)	63 (55)	
**AUDIT total score, Mean (SEM)**	14.34 (0.93)	14.02 (1.14)	14.91 (1.61)	*p* = 0.64	14.25 (1.60)	14.39 (1.14)	*p* = 0.94

Continuous and categorical variables are compared using independent samples t-test and chi-squared test, respectively. *AIMs,* Ancestry informative markers; *AUDIT,* Alcohol Use Disorder Identification Test; *BMI,* Body Mass Index; *GLP-1R,* Glucagon-Like Peptide-1 Receptor; *TLFB,* TimeLine FollowBack.

### Imaging genetic findings

**Figure 1:**
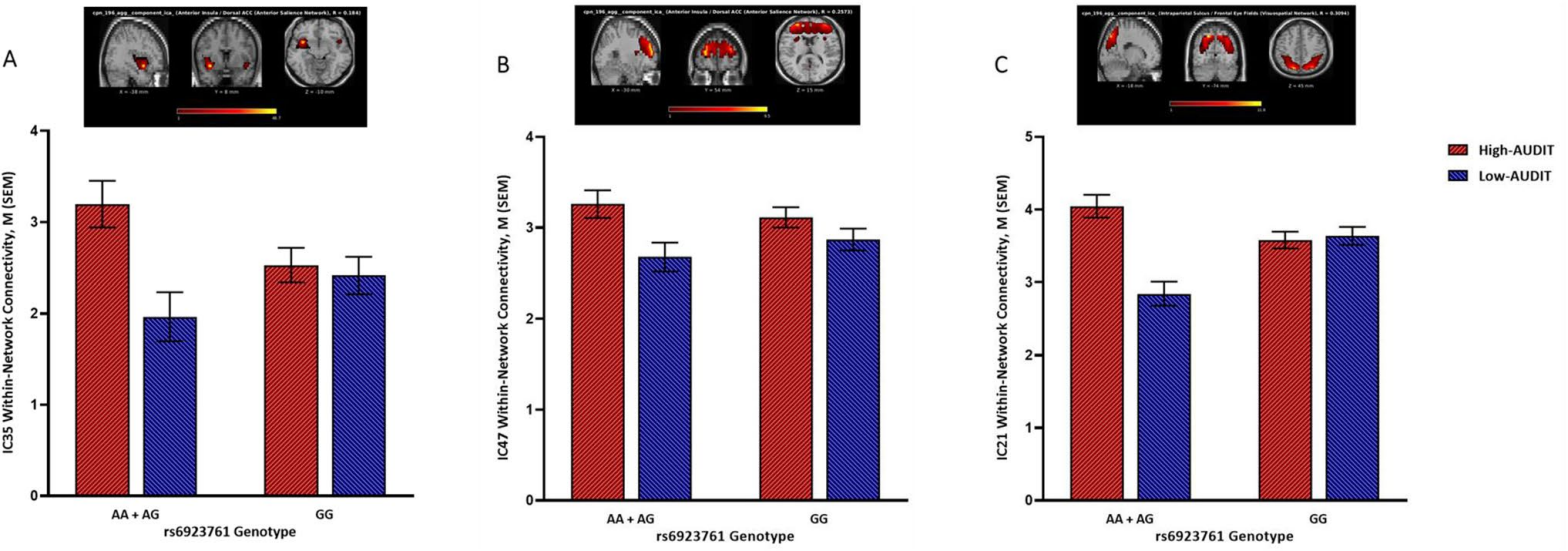
Normalized within-network connectivity estimates for significant interactions between AUDIT group and rs6923761 genotype. Top panels (brain images) include the component T-map extracted during the ICA processing step; colors represent T-max values. High-AUDIT: AUDIT total score ≥ 8 (n = 96); Low-AUDIT: AUDIT total score < 8 (*n* = 85). A is the risk allele; A-allele carriers: AA + AG (n = 65); Non-A-allele carriers: GG (*n* = 116). IC35 (insula; **A**) and IC47 (frontal/acc/putamen; **B**) were mapped onto the anterior salience network; IC21 (insula/occipital/mpfc; **C**) was mapped onto the visuospatial network.

For rs6923761, three ICs (21, 35, 47) showed significant genotype × AUDIT interaction effects on within-network connectivity. IC35 and IC47 were mapped onto the anterior salience network and IC21 was mapped onto the visuospatial network (Table S1). Normalized estimates of connectivity within these significant components are depicted in Fig. 1. Overall, in the group carrying the risk allele (AA + AG), high AUDIT, compared to low AUDIT, was associated with stronger within-network connectivity, but a weaker or no association was found in the protected group (GG) (Fig. 1). Results of the full MANCOVA model are depicted in Figure S1.

**Figure 2:**
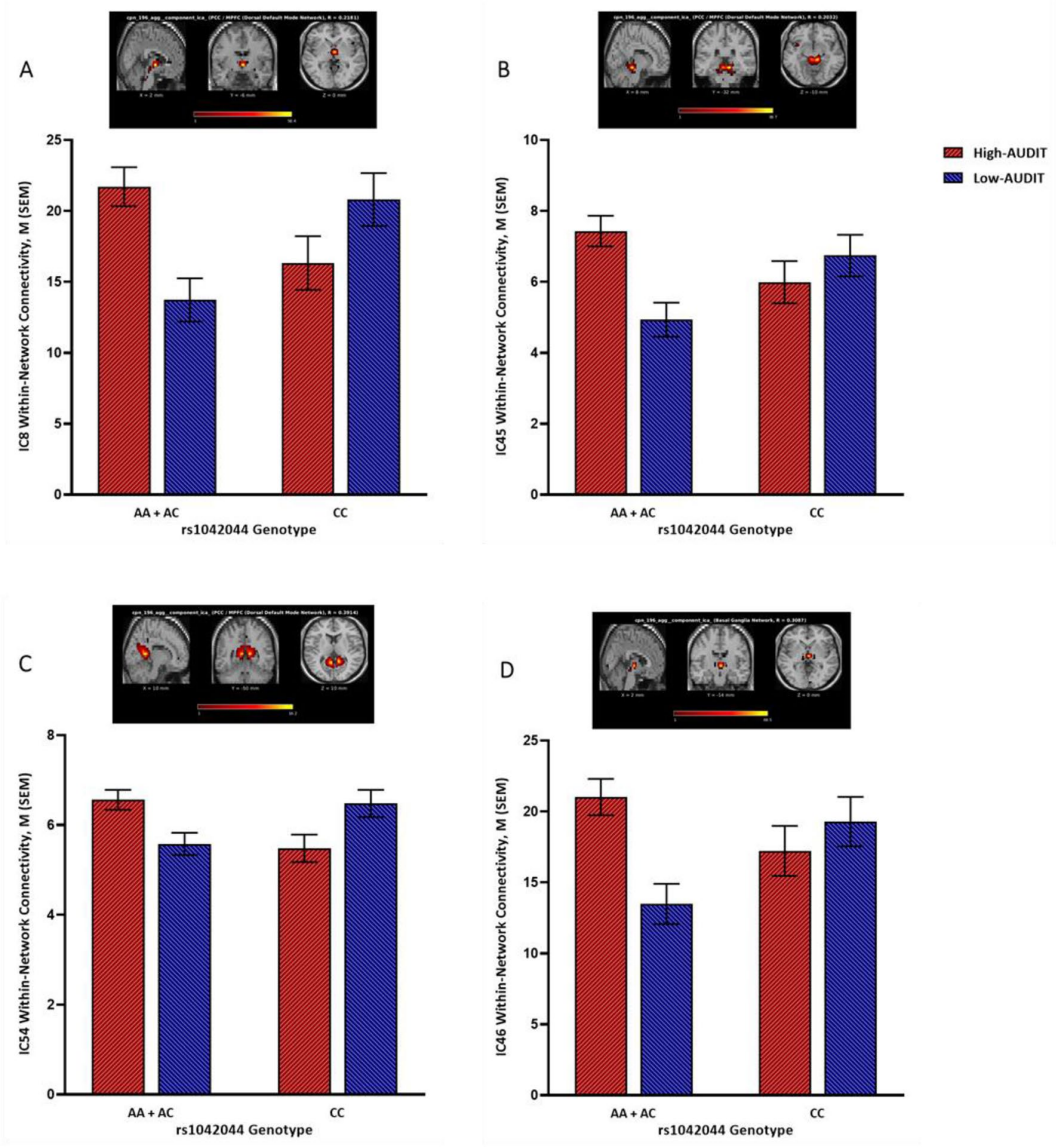
Normalized within-network connectivity estimates for significant interactions between AUDIT group and rs1042044 genotype. Top panels (brain images) include the component T-map extracted during the ICA processing step; colors represent T-max values. High-AUDIT: AUDIT total score ≥ 8 (*n* = 96); Low-AUDIT: AUDIT total score < 8 (*n* = 85). A is the risk allele; A-allele carriers: AA + AC (*n* = 114); Non-A-allele carriers: CC (*n* = 67). IC8 (hypothalamus/brainstem; **A**), IC45 (thalamus/3rd ventricle; **B**), and IC54 (pcc/tpj/occipital/mpfc; **C**) were mapped onto the dorsal default mode network; IC46 (thalamus/brainstem; **D**) was mapped onto the basal ganglia network.

For rs1042044, four ICs (8, 45, 46, 54) showed significant genotype × AUDIT interaction effects on within-network connectivity. IC8, IC45, and IC54 were mapped onto the dorsal default mode network and IC46 was mapped onto the basal ganglia network (Table S1). Normalized estimates of connectivity within these significant components are depicted in Fig. 2. Overall, in the group carrying the risk allele (AA + AC), high AUDIT, compared to low AUDIT, was associated with stronger within-network connectivity, but an opposite association was found in the protected group (GG) (Fig. 2). Results of the full MANCOVA model are depicted in Figure S2.

No significant genotype × AUDIT interaction effects on between-network connectivity were found (Figures S3 and S4).

## Discussion

The goal of this human study was to investigate the relationship between the GLP-1 system and alcohol-related outcomes by exploring the interaction between genetic variation at the GLP-1R and severity of alcohol use on resting-state brain functional connectivity. The most important findings, indicated by multiple significant ICs, showed stronger within-network connectivity in the anterior salience network and the default mode network among individuals with high versus low severity of alcohol use (as indicated by AUDIT score), in the groups carrying the risk allele for each of the two SNPs that were selected a priori for this analysis. Similar findings were also shown in the visuospatial network and the basal ganglia network. Albeit preliminary and in need of replication, these results suggest possible brain regions and circuits involved in the link between the GLP-1 system and alcohol seeking/consummatory behaviors—an area of research that has recently gained more attention, to the point that targeting the GLP-1 system is under investigation as a potential pharmacotherapeutic approach for AUD^6,52^.

Imaging genetics is a growing area of research that has already informed the neurobiological underpinnings of addictive behaviors and AUD^53–56^. It is important to note that this study focused on two specific SNPs of the GLP-1R gene that were selected a priori, given their putative functional relevance, as they lead to amino-acid substitutions, changes in the GLP-1R structure and function, and various metabolic and endocrine consequences^36–43^. The first SNP, rs6923761, results in an amino-acid substitution of glycine, which is a nonpolar molecule, to serine, a polar molecule, at position 168. Due to this change in polarity, the presence of the variant allele changes the structure and folding of the GLP-1R and, therefore, would likely decrease the activity and responsiveness of the receptor to the GLP-1 peptide. An example of how these molecular changes may translate into clinically relevant outcomes was shown in our previous study, where we found significantly higher alcohol self-administration in a controlled laboratory setting among carriers of the variant allele, compared to the protected group^30^. The other GLP-1R SNP studied here, rs1042044, also leads to an amino-acid substitution of leucine to phenylalanine at position 260, with potential changes in the structure, folding, and function of the GLP-1R. The variant allele of this SNP was found to be associated with a significant increase in morning cortisol levels^40^, which is particularly relevant, given the interaction between GLP-1 and the HPA axis^17–19^ and the established role of stress hormones in the pathophysiology of AUD^57^. Of note, the results described here indicate potential important associations but do not establish causality. Therefore, whether the differences in brain functional connectivity observed in this study are directly related to changes in the GLP-1R, downstream effects such as other neuroendocrine changes, or both, remains unknown and should be further studied in future research.

For rs6923761, two of the three significant ICs (IC 35: insula; IC 47: frontal lobe, anterior cingulate cortex, and putamen) were mapped onto the anterior salience network, which is particularly noteworthy, as this network has been linked to AUD in previous research. The anterior salience network is mostly comprised of the anterior insula and the anterior cingulate cortex, which work together to guide behaviors based on internal stimuli and visual stimuli in extrapersonal space. In this system, the insula functions as the “integral hub” by mediating information flow across other brain networks involved in attention and cognition^58^. In one relevant study, Grodin and colleagues compared neuroimaging and self-reported measures from 60 individuals with alcohol dependence and 49 healthy controls. They found smaller volumes of structures involved in the anterior salience network, namely anterior insula and anterior cingulate cortex, among individuals with alcohol dependence, and these structural measures were negatively correlated with measures of impulsivity and compulsivity^59^. Other studies have also found that cognitive impairments observed in individuals with AUD might be linked to structural and/or functional abnormalities in the salience network^60^. It is important to note that the GLP-1 system has been shown to play an important role in cognitive processes and the GLP-1R is expressed in brain regions involved in executive function, such as hippocampus and prefrontal cortex^9,61,62^. Preclinical studies have also linked changes in GLP-1R expression to cognitive function. In one study, for example, GLP-1R knockout mice showed considerable learning deficits, while overexpression of the GLP-1R led to improvement in learning and memory^63^. It is plausible to hypothesize that the salience network and cognitive function contribute, at least in part, to the link between the GLP-1 system and alcohol-related outcomes.

The other significant IC related to rs6923761 (IC 21: insula, occipital lobe, and medial prefrontal cortex) was mapped onto the visuospatial network. This network controls cognitive processes necessary to identify and analyze space in a visual form, and tends to involve connections between the precuneus, posterior parietal cortex, middle frontal gyrus, and parts of the parieto-occipital cortex^64,65^. The visuospatial network has been linked to cue reactivity in the context of AUD. In one study, Fukushima and colleagues found stronger fMRI BOLD response in the left precuneus among individuals with AUD, compared to controls, when they were exposed to images depicting drinking alcohol, and opposite results when exposed to images of drinking juice^66^. Another study looked at the connection between the visuospatial network and parietal regions, and found weaker resting-state connectivity to the visuospatial network in individuals with AUD, compared to controls, implying potential deficits in memory encoding and insufficient visuospatial information for well-controlled movements^67^. Of note, the GLP-1R is widely expressed in the hippocampus, and some evidence suggests that GLP-1 system in the brain may also play a role in regulating visuospatial memory^68^.

For rs1042044, three of the four significant ICs (IC 8: hypothalamus and brainstem; IC 45: thalamus and third ventricle; IC 54: posterior cingulate cortex, temporoparietal junction, occipital lobe, and medial prefrontal cortex) were mapped onto the dorsal default mode network. The default mode network is a significant network that is impacted by alcohol and primarily consists of the medial prefrontal cortex, posterior cingulate cortex, lateral and medial temporal lobes, and posterior interior parietal lobule. These structures work synergistically to regulate autobiographical planning, like imagining personal future events and other self-initiated mental activities^69^. Kamarajan and colleagues used EEG to investigate within-network connectivity and found hyperconnectivity in the default mode network in individuals with AUD, compared to healthy controls, which was hypothesized to be linked with increased impulsivity in these individuals^70^. Other studies, however, have found decreased within-network connectivity in the default mode network as a result of alcohol use. Fang and colleagues conducted a randomized, crossover, placebo-controlled study with intravenous alcohol infusion (target BrAC = 0.8 g/kg) in 37 heavy-drinking individuals and found that resting-state, within-network connectivity of the default mode network significantly decreased during alcohol exposure. This effect was more prominent in individuals who reported higher craving for alcohol^71^. Another study also found lower functional connectivity within the default mode network in individuals with AUD, compared to healthy controls^72^. Few studies have looked at the relationship between GLP-1 signaling and the default mode network. One study compared the effects of GLP-1R blockade before and after Roux-en-Y gastric bypass (RYGB) surgery and found increased functional connectivity in the default mode network post-RYGB, compared to pre-RYGB^73^. Collectively, these findings suggest that both alcohol and GLP-1 interact with the default mode network in the brain. However, more studies are needed to disentangle and characterize this link.

The other significant IC related to rs1042044 (IC 46: thalamus and brainstem) was mapped onto the basal ganglia network, which plays a key role in motor control, motor learning, executive functions, and emotions^74^. Preclinical evidence indicates that chronic alcohol exposure may affect the basal ganglia network, and dysfunctions in this network may contribute to alcohol-related cognitive impairment^75^. Previous research also suggests that the basal ganglia network plays a role in alcohol craving and loss of control over alcohol consumption^76^. The GLP-1 system also interacts with the basal ganglia network. As an example, Mora and colleagues showed that perfusion of GLP-1 (7–36) amide led to a selective increase in the extracellular levels of glutamine and glutamic acid in the basal ganglia of rats^77^.

Consistent with Allen and colleagues^50^, we used a multivariate approach for analyzing resting-state data in this study, reducing the total number of statistical tests performed. In this hierarchical approach, backward selection is a key step, which tests whether each predictor in the model explains variability in the multivariate response, using a MANCOVA. The two SNPs were analyzed separately. Adjustment for multiple comparisons (i.e., two separate tests) was not performed at this level, mainly because our analytic approach was selective and conservative enough and overcontrolling could mask potentially relevant signals^78^, especially given that clinical literature on the link between the GLP-1 system and AUD is very limited. The two SNPs were selected a priori for this study based on previous literature in AUD and other fields, as well as their putative functional consequences. Furthermore, we a priori selected a 2 × 2 (genotype × AUDIT) interaction as our only outcome of interest in order to detect the most clinically relevant signal, if any, while minimizing the risk of spurious findings.

The present study had several limitations that must be acknowledged. First and foremost, the sample size was relatively small. Albeit selected a priori and based on functional relevance, only two GLP-1R SNPs were studied, and only a dominant model was applied, given the small number in each cell. Future studies with larger and more diverse samples can include an additive model to see whether the presence of one or two variant alleles may differentially impact brain functional connectivity. While both SNPs lead to amino-acid substitutions and putative changes in the GLP-1R structure and function, lack of overlap between the networks implicated by the two SNPs suggest that they may not be associated with similar biological changes—an aspect that is beyond the scope of the present study. It is also possible that the two genotyped markers are simply acting as surrogates for unknown functional variants, although the linkage disequilibrium (LD) structure at GLP-1R gene in HapMap populations suggests that these occult functional loci would be within the GLP-1R gene, because the LD blocks not extend into neighboring genes. The two variants are located on different haplotype backgrounds, and therefore could either be acting through differing mechanisms, or acting as surrogates for unknown functional loci that function through differing mechanisms. Accordingly, whether the effects observed are directly driven by changes in the GLP-1 system, other neurobiological pathways, or both remains unknown and should be explored in future research. Given that this study was the first one of its kind, we applied a whole brain approach that divided the brain agnostically and examined all resulting components. Future research may choose to use a more targeted, seed-based connectivity approach guided by the findings of this study. Considering the setting of the study, we were not able to standardize nutrition, activities, and other factors prior to the brain fMRI scan. Another important consideration is differences in minor allele frequencies across racial/ethnic groups from diverse ancestral backgrounds. The minor allele of rs6923761 had a low frequency among Black than White individuals, while the numbers were more balanced for rs1042044 (Table 1). Although we controlled for AIMs score and, therefore, current analyses are likely not influenced by this factor, future studies with larger sample sizes may conduct subgroup analyses, e.g., among those from African versus European ancestral background. Finally, while AUDIT is a well-validated and widely used measure, it relies exclusively on self-reported data and, therefore, may be subject to report bias. We dichotomized the AUDIT score, mainly due to our small sample size and to examine a 2 × 2 (genotype and AUDIT) interaction. Future studies with more power should consider including AUDIT score as a continuous variable to examine the full spectrum of alcohol use severity.

In conclusion, the present study found that genetic variation at the GLP-1R was differentially associated with brain functional connectivity in individuals with low versus high severity of alcohol use. Specifically, the presence of the variant alleles was associated with stronger within-network connectivity in those with high versus low severity of alcohol use. Significant findings in the salience and default mode networks are particularly relevant, given their role in the neurobiology of AUD and addictive behaviors. Future studies should investigate the specific mechanisms underlying these effects and how they may inform ongoing work to target the GLP-1 system as a potential new pharmacotherapy for AUD.

## Supplementary Information

Supplementary Information.

## Data Availability

The datasets used and analyzed for the current study are available from the corresponding author upon reasonable request.
