# Supplementary material for: Differential association between the GLP1R gene variants and brain functional connectivity according to the severity of alcohol use
Source: Sci Rep. 2022 Jul 29;12:13027. doi: 10.1038/s41598-022-17190-3 (PMC9338323; doi:10.1038/s41598-022-17190-3)
Supplement: Supplementary file 1 — Supplementary Information. [file 41598_2022_17190_MOESM1_ESM.docx]

**Supplementary Information**

Farokhnia M, Fede SJ, Grodin EN, Browning BD, Crozier ME, Schwandt ML, Hodgkinson CA, Momenan R, Leggio L. Differential Association between the *GLP1R* Gene Variants and Brain Functional Connectivity According to the Severity of Alcohol Use.

**Table of Contents:**

- **Table S1.** List of the final 51 independent components (ICs) and their respective labels
- **Table S2.** Demographic characteristics of the full sample and stratified by AUDIT group
- **Figure S1.** Results from the spatial map intensity (within-network connectivity) MANCOVA model with rs6923761
- **Figure S2.** Results from the spatial map intensity (within-network connectivity) MANCOVA model with rs1042044
- **Figure S3.** Results from the functional network connectivity MANCOVA model with rs6923761
- **Figure S4.** Results from the functional network connectivity MANCOVA model with rs1042044

**Table S1.** List of the final 51 independent components (ICs) and their respective labels

| **IC** | **Description** | **Label** |
| --- | --- | --- |
| 35 | insula | Anterior Insula / Dorsal ACC (Anterior Salience Network) |
| 47 | frontal/acc/putamen | Anterior Insula / Dorsal ACC (Anterior Salience Network) |
| 48 | insula/acc/pcc | Anterior Insula / Dorsal ACC (Anterior Salience Network) |
| 50 | insula/frontal/pcc | Anterior Insula / Dorsal ACC (Anterior Salience Network) |
| 5 | thalamus | Posterior Insula (Posterior Salience Network) |
| 28 | insula | Posterior Insula (Posterior Salience Network) |
| 37 | cerebellum/thalamus/ba22 | Posterior Insula (Posterior Salience Network) |
| 39 | insula/pcc/ba44/40/temporal | Posterior Insula (Posterior Salience Network) |
| 49 | ba9/7/31/8/39/insula/temporal | Posterior Insula (Posterior Salience Network) |
| 16 | caudate/putamen/thalamus/brainstem | Basal Ganglia Network |
| 27 | caudate/putamen | Basal Ganglia Network |
| 46 | thalamus/brainstem | Basal Ganglia Network |
| 44 | ba9/ba6/temporal/visual association/cerebellum | Left DLPFC / Parietal (Left Executive Control Network) |
| 8 | hypothalamus/brainstem | PCC / MPFC (Dorsal Default Mode Network) |
| 10 | cerebellum/occipital | PCC / MPFC (Dorsal Default Mode Network) |
| 13 | frontal/dlpfc/cerebellum/some pcc | PCC / MPFC (Dorsal Default Mode Network) |
| 19 | thalamus | PCC / MPFC (Dorsal Default Mode Network) |
| 31 | pcc/tpj/temproal pole | PCC / MPFC (Dorsal Default Mode Network) |
| 32 | thalamus/3rd ventricle/insula/brainstem | PCC / MPFC (Dorsal Default Mode Network) |
| 36 | acc/ofc/pcc | PCC / MPFC (Dorsal Default Mode Network) |
| 45 | thalamus/3rd ventricle | PCC / MPFC (Dorsal Default Mode Network) |
| 54 | pcc/tpj/occipital/mpfc | PCC / MPFC (Dorsal Default Mode Network) |
| 14 | visual association/parahippocampal/cerebellum | Retrosplenial Cortex / Medial Temporal Lobe  (Ventral Default Mode Network) |
| 29 | pcc/precuneus | Retrosplenial Cortex / Medial Temporal Lobe  (Ventral Default Mode Network) |
| 51 | pcc/precuneus/acc/caudate/tpj | Retrosplenial Cortex / Medial Temporal Lobe  (Ventral Default Mode Network) |
| 52 | ba8/caudate/cerebellum | Retrosplenial Cortex / Medial Temporal Lobe  (Ventral Default Mode Network) |
| 53 | pcc | Retrosplenial Cortex / Medial Temporal Lobe  (Ventral Default Mode Network) |
| 62 | cerebellum/pcc/insula | Retrosplenial Cortex / Medial Temporal Lobe  (Ventral Default Mode Network) |
| 7 | occipital/cerebellum, some noise | Precuneus Network |
| 18 | precuneus/cerebellum | Precuneus Network |
| 3 | occipital/cerebellum | Primary Visual Network |
| 11 | occipital/thalamus | Primary Visual Network |
| 30 | visual association/ba19 | Primary Visual Network |
| 22 | occipital/cerebellum | Higher Visual Network |
| 9 | posterior insula | Intraparietal Sulcus / Frontal Eye Fields  (Visuospatial Network) |
| 20 | insula/frontal/tpj/frontal eye field/ofc | Intraparietal Sulcus / Frontal Eye Fields  (Visuospatial Network) |
| 21 | insula/occipital/mpfc | Intraparietal Sulcus / Frontal Eye Fields  (Visuospatial Network) |
| 26 | cerebellum/fusiform/thalamus | Intraparietal Sulcus / Frontal Eye Fields  (Visuospatial Network) |
| 43 | pcc/ba8/tpj/ba10/cerebellum/ba22 | Intraparietal Sulcus / Frontal Eye Fields  (Visuospatial Network) |
| 12 | post central/parahippocampla/primary sensory | Sensorimotor Network |
| 17 | posterior cingulate / some ventricles / cerebellum | Sensorimotor Network |
| 33 | primary sensory/tpj/temporal/insula/lateral frontal/cerebellum | Sensorimotor Network |
| 34 | caudate/putamen/ba19/visual association/parahippocampal | Sensorimotor Network |
| 38 | cerebellum/caudate/parahippocampal/ba22 | Sensorimotor Network |
| 55 | caudate/cerebellum/4thventricle | Sensorimotor Network |
| 56 | insula/pcc/primary sensory/ba7 | Sensorimotor Network |
| 61 | cerebellum/visual association/thalamus | Sensorimotor Network |
| 6 | bilateral temporoparietal | Auditory Network |
| 24 | insula/tpj/frontal | Language Network |
| 25 | tpj/frontal/primvisual/thalamus | Language Network |
| 60 | insula/frontal/temporal | Language Network |

**Table S2.** Demographic characteristics of the full sample and stratified by AUDIT group

|  |  | **AUDIT** | | |
| --- | --- | --- | --- | --- |
|  | **Full Sample**  **(n = 181)** | **Low, < 8**  **(n = 85)** | **High, ≥ 8**  **(n = 96)** | **Statistics** |
| **Age, years, Mean (SEM)** | 40.57 (0.90) | 37.88 (1.23) | 43.29 (1.25) | *p* = 0.003 |
| **Sex, n (%)**  - Male  - Female | 104 (57)  77 (43) | 37 (43)  48 (57) | 67 (70)  29 (30) | *p* < 0.001 |
| **Years of education, Mean (SEM)** | 14.95 (0.23) | 16.28 (0.34) | 13.77 (0.28) | *p* < 0.001 |
| **BMI, Kg/m^2^, Mean (SEM)** | 26.72 (0.36) | 26.49 (0.56) | 26.91 (0.47) | *p* = 0.56 |
| **Smoking status, n (%)**  - Smoker  - Non-smoker | 47 (26)  134 (74) | 1 (1)  84 (99) | 46 (48)  50 (52) | *p* < 0.001 |
| **AIMs score, Mean (SEM)**  - Europe  - Africa | 0.50 (0.02)  0.37 (0.02) | 0.48 (0.04)  0.34 (0.04) | 0.52 (0.03)  0.39 (0.04) | *p* = 0.52  *p* = 0.37 |
| **Race, n (%)**  - Black  - White  - Asian  - Multiple races  - Unknown | 76 (42)  77 (42)  10 (6)  9 (5)  9 (5) | 32 (38)  36 (42)  8 (9)  4 (5)  5 (6) | 44 (46)  41 (43)  2 (2)  5 (5)  4 (4) | *p* = 0.24 |
| **90-day TLFB, Mean (SEM)**  - Average drinks per day  - Heavy drinking days | 7.91 (0.67)  30.56 (2.68) | 1.86 (0.10)  1.71 (0.80) | 12.38 (0.93)  56.11 (3.25) | *p* < 0.001  *p* < 0.001 |
| **AUDIT total score, Mean (SEM)** | 14.34 (0.93) | 2.58 (0.20) | 24.75 (0.80) | *p* < 0.001 |

**Note:** Demographic characteristics are presented in the full sample and separately across the two AUDIT groups (low: < 8, high: ≥ 8). Continuous and categorical variables are compared using independent samples t-test and chi-squared test, respectively. *AIMs*, Ancestry informative markers; *AUDIT*, Alcohol Use Disorder Identification Test; *BMI*, Body Mass Index; *TLFB*, TimeLine FollowBack.

**Figure S1.** Results from the spatial map intensity (within-network connectivity) MANCOVA model with rs6923761


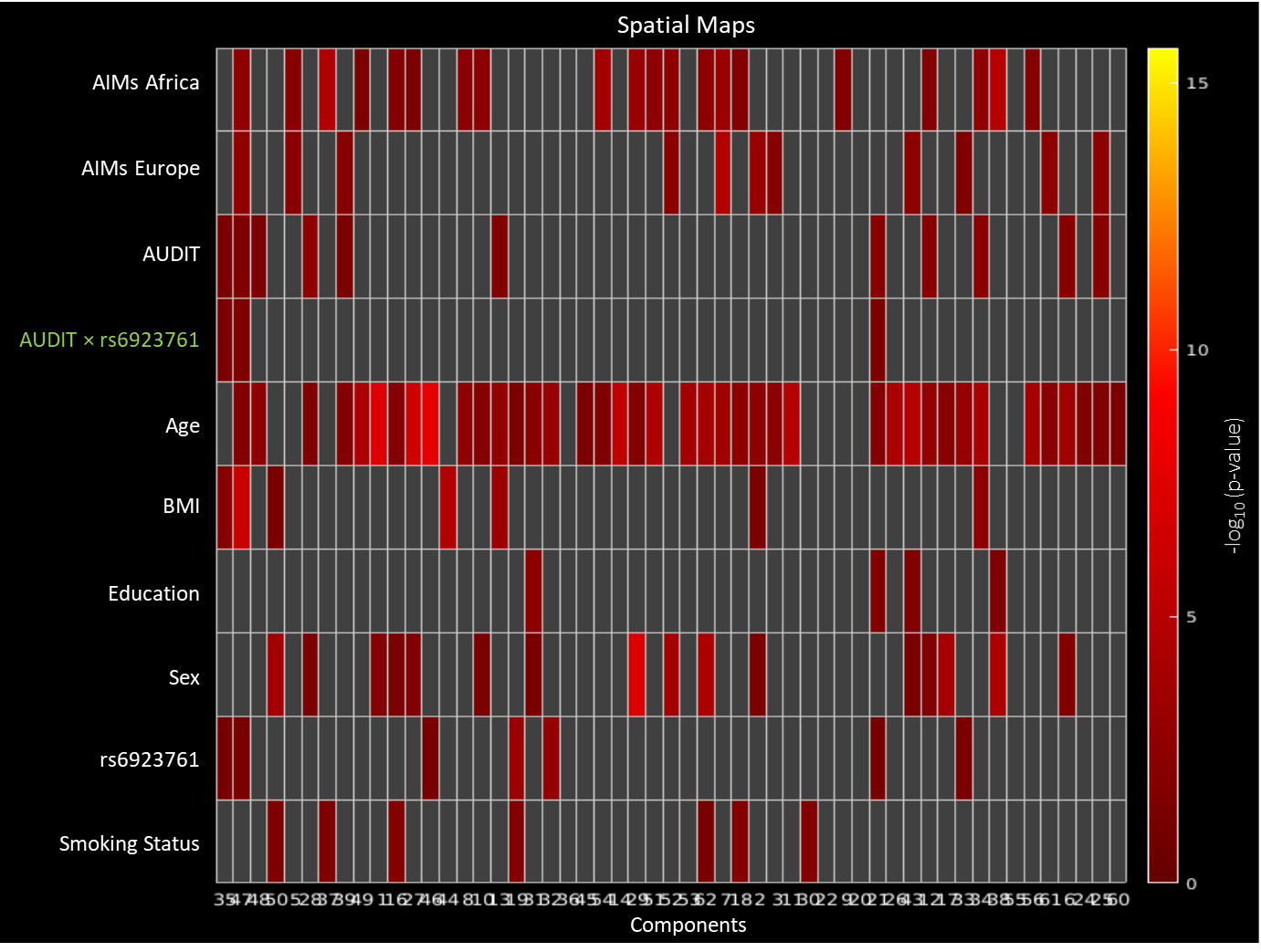


**Note:** Gray cells indicate terms that were removed from the full model during backward selection process. Results are depicted in -log_10_(p) units following the gradient bar.

**Figure S2.** Results from the spatial map intensity (within-network connectivity) MANCOVA model with rs1042044


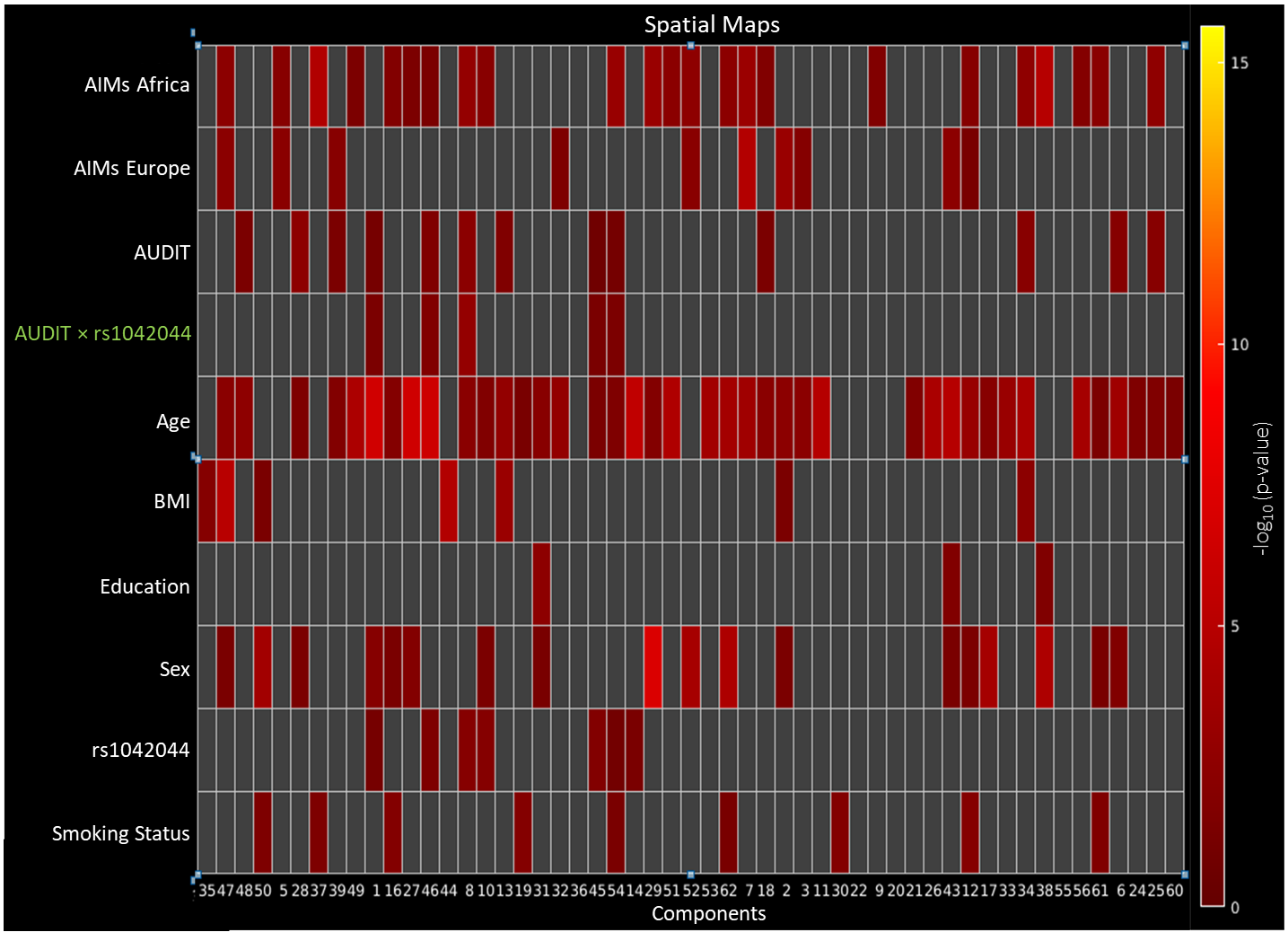


**Note:** Gray cells indicate terms that were removed from the full model during backward selection process. Results are depicted in -log_10_(p) units following the gradient bar.

**Figure S3.** Results from the functional network connectivity MANCOVA model with rs6923761


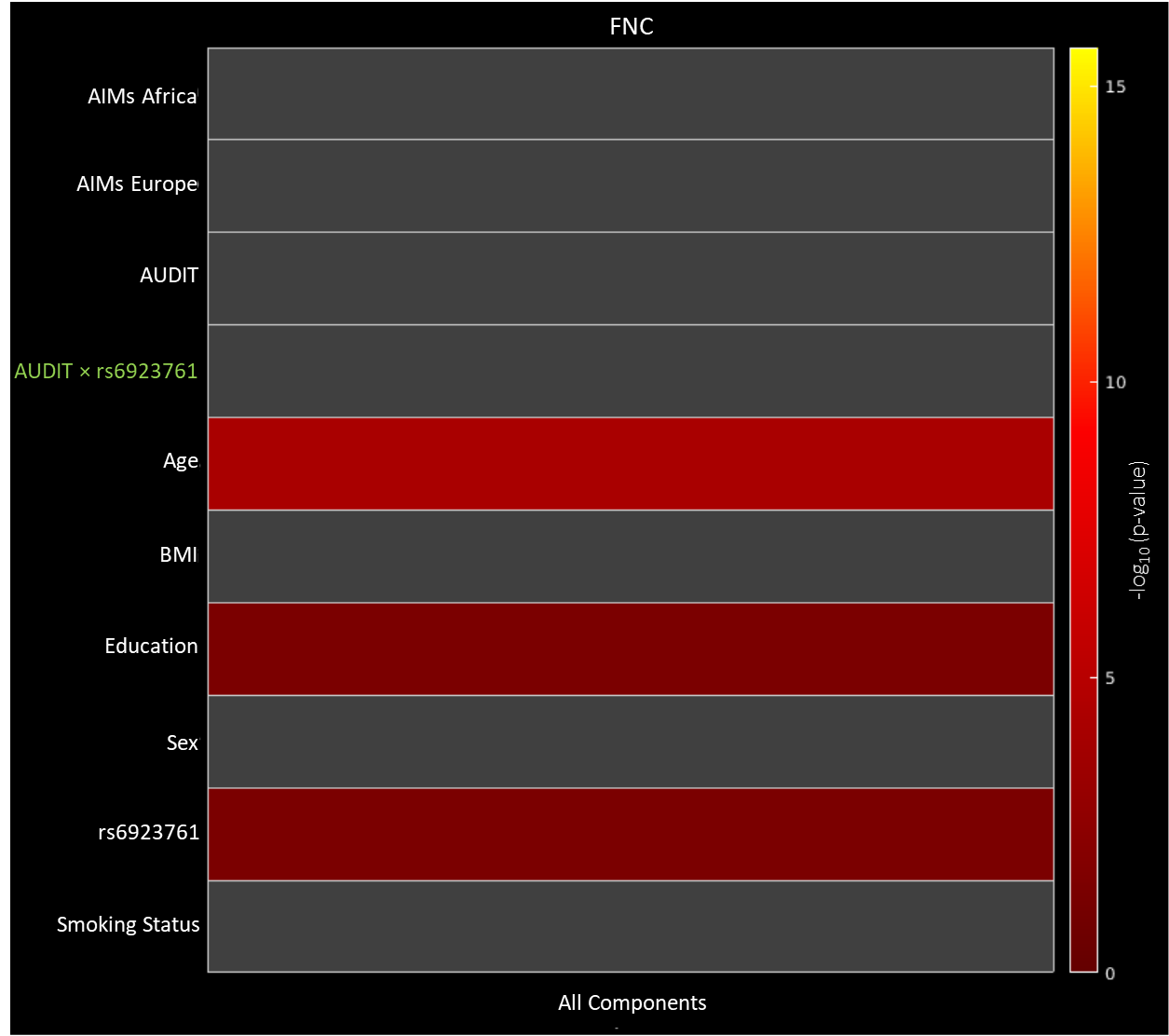


**Note:** Gray cells indicate terms that were removed from the full model during backward selection process. Results are depicted in -log_10_(p) units following the gradient bar.

**Figure S4.** Results from the functional network connectivity MANCOVA model with rs1042044


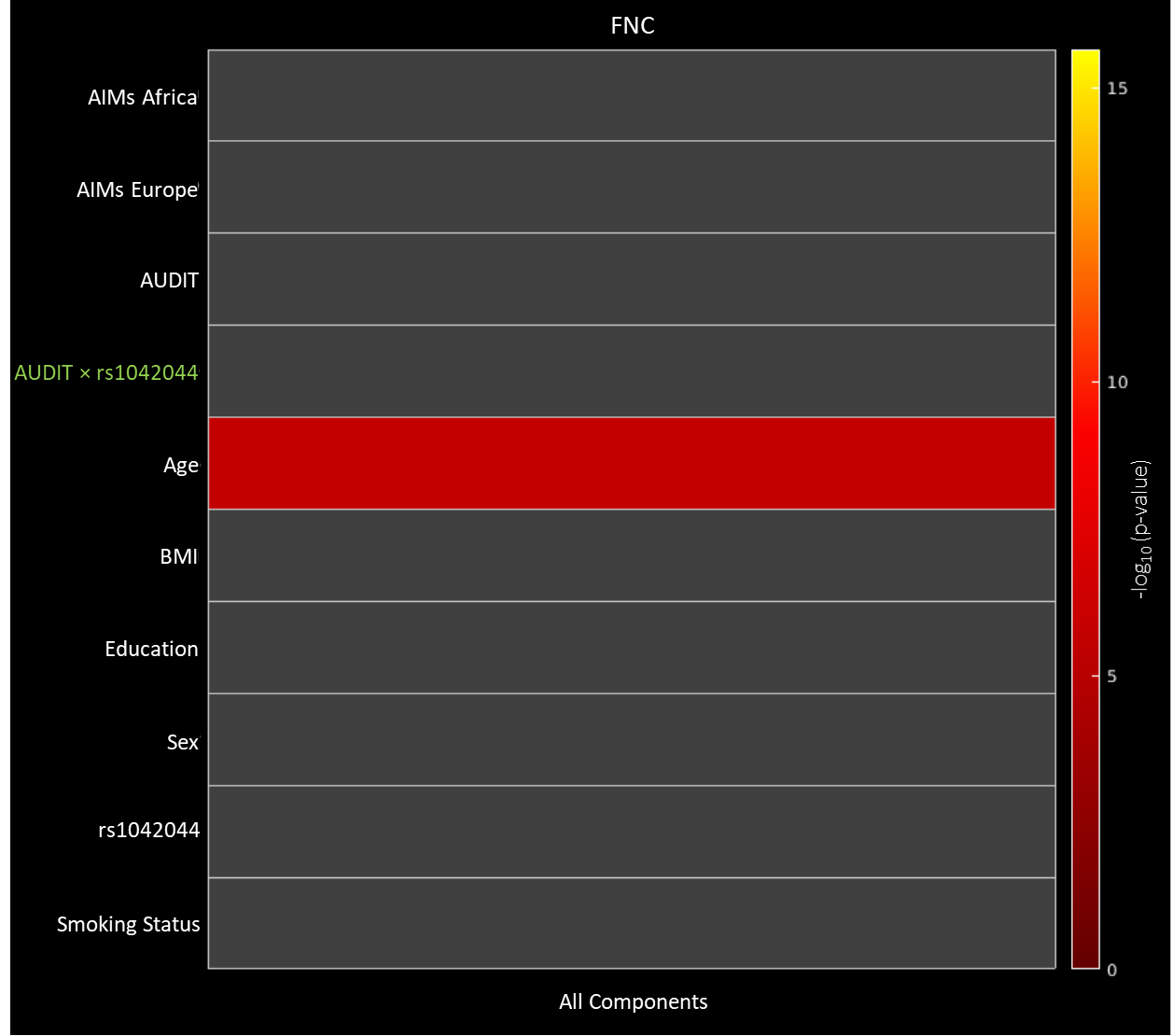


**Note:** Gray cells indicate terms that were removed from the full model during backward selection process. Results are depicted in -log_10_(p) units following the gradient bar.
